# Fear learning and generalization in youth with early-stage transdiagnostic psychiatric symptoms and the impact of acute exercise

**DOI:** 10.3389/fpsyt.2025.1657470

**Published:** 2025-09-19

**Authors:** Lise Jennen, Celine Samaey, Zhiling Qiao, Victor Mazereel, Kristof Vansteelandt, Davy Vancampfort, Ruud van Winkel

**Affiliations:** ^1^ Center for Clinical Psychiatry, Department of Neurosciences, KU Leuven, Leuven, Belgium; ^2^ Brain and Cognition, Laboratory for Biological Psychology, KU Leuven, Leuven, Belgium; ^3^ School of Psychology, Shandong Normal University, Ji’an, China; ^4^ Integrated Psychiatric Center Openbaar Psychiatrisch Zorgcentrum (OPZ), Geel, Belgium; ^5^ University Psychiatric Center, KU Leuven, Leuven-Kortenberg, Belgium; ^6^ Adapted Physical Activity and Psychomotor Rehabilitation, Department of Rehabilitation Sciences, KU Leuven, Leuven, Belgium

**Keywords:** fear conditioning, depression, anxiety, psychosis, adolescents, young adults, acute exercise intervention

## Abstract

**Background:**

Adolescence and early adulthood represent critical periods for the emergence of psychiatric symptoms, often spanning multiple symptom dimensions. Alterations in fear learning and generalization are implicated in anxiety-related disorders, yet research on these processes in youth with early-stage transdiagnostic psychiatric symptoms remains limited.

**Methods:**

This study investigated fear learning and generalization in youth aged 16–24 years with transdiagnostic psychiatric symptoms (anxiety, depressive, and psychotic), as indexed by US expectancy ratings. Additionally, considering the modulatory impact of exercise on memory processes, we explored the effects of a 10-minute moderate-intensity exercise intervention using a randomized between-subject design.

**Results:**

Contrary to hypotheses, the symptom group did not show impaired threat–safety discrimination or overgeneralization of fear. However, they exhibited elevated overall threat expectancy during generalization, suggesting that a bias in threat expectancy could represent an early vulnerability in threat processing. Dimensional analyses point to subtle symptom-specific differences in generalization patterns, underscoring the importance of modeling continuous symptom severity alongside group-based comparisons. No significant effects of acute exercise on fear acquisition or generalization were observed.

**Conclusion:**

These findings highlight early alterations in threat processing in youth with early-stage psychiatric symptoms. Future research should investigate symptom-specific patterns in fear generalization, track their longitudinal development, and refine exercise interventions to effectively modulate fear processing.

## 1 Introduction

Mental health disorders typically arise in the context of complex genetic and environmental factors and are marked by multiple alterations at the level of cognitive processing. Among these are alterations in threat processing, such as fear learning and fear generalization (1). Fear learning, typically examined using a classical conditioning task, involves pairing a neutral stimulus with an aversive unconditioned stimulus (US) to create a conditioned stimulus (CS+) that signals danger, while another stimulus that is never paired with the US becomes the safety cue (CS-). Fear generalization occurs when fear extends from a conditioned stimulus to similar cues (2), which can be assessed by presenting generalization stimuli (GSs) that vary in similarity to the CS+. While this process is essential for survival, it can become maladaptive when fear overgeneralizes to similar neutral cues, potentially contributing to anxiety disorders and other forms of psychopathology (3).

Mental health symptoms often first emerge during adolescence and early adulthood (4). This period is characterized by early-stage, mixed symptom presentations that may not meet full diagnostic criteria (5), yet can signal increased risk for later psychopathology. Despite its clinical relevance, research on fear learning and generalization has left this important developmental window understudied. Investigating altered threat processing in this population is crucial for further understanding relevant mechanisms underlying the emergence of mental health disorders.

Previous research on threat processing has primarily focused on anxiety and stress-related disorders, such as generalized anxiety disorder and social anxiety disorder, where increased fear acquisition and overgeneralization of fear to ambiguous stimuli have been consistently demonstrated (2, 3, 6–8). Importantly, even at the subclinical level, anxiety symptoms have been linked to altered threat processing, with impaired discrimination and heightened generalization predicting more anxiety over time (9), and fear generalization correlating with anxious personality (10). However, alterations in threat processing may not be exclusive to the anxiety spectrum. For instance, reduced CS discrimination without clear generalization effects has been observed in individuals with major depressive disorder (11), while overgeneralization of fear has been associated with anhedonia, a core symptom of depression (12). Moreover, both depressive and psychotic symptoms have been linked to an attentional bias toward interpreting neutral stimuli as threatening (1), underscoring the potential transdiagnostic relevance of aberrant threat processing.

Early-stage psychiatric symptoms in young people represent a major contributor to health-related disability (13), underscoring the urgent need for accessible early intervention strategies. Exercise has gained increasing attention given its broad cognitive benefits and potential to influence affective learning processes (14, 15). Most exercise studies have focused on fear conditioning or fear extinction, but fear generalization has remained largely unexamined. Acute bouts of exercise can modulate neurobiological systems implicated in fear learning, including neurotransmitter function, HPA axis activity, and hippocampus-dependent memory processes (16, 17). Given the critical role of the hippocampus in regulating fear generalization (18), exercise may offer a promising means of modulating this process.

In this study, we used a classical fear conditioning paradigm to investigate fear learning and generalization in youth aged 16–24. To capture the heterogeneity and pluripotent nature typical of early-stage psychiatric symptom presentation (19, 20), we adopted a transdiagnostic approach focused on depressive, anxiety, and psychotic symptom dimensions rather than categorical diagnoses. We combined a group-level comparison with a dimensional analysis. First, a group with early-stage transdiagnostic symptoms was compared to a healthy control group. It was hypothesized that the symptom group would exhibit reduced threat-safety discrimination during acquisition, particularly reflected by increased fear responding to the safety cue, and increased fear generalization, defined as transfer of fear to the similar stimuli with trial-by-trial US expectancy ratings as our main outcome measure. Within the symptom group, a dimensional analysis was subsequently conducted to examine whether specific alterations in threat processing are related to individual symptom dimensions. This approach acknowledges the clinical relevance of identifying early-stage group differences while also aligning with recent advances towards dimensional models of psychopathology, which suggest that fear learning alterations vary continuously with symptom severity, rather than categorically (21). We hypothesized that anxiety symptoms would show the strongest association with alterations in threat processing, while depressive and psychotic symptom dimensions would show more subtle or distinct patterns of association.

Additionally, we explored whether a 10-minute bout of moderate-intensity exercise could influence fear acquisition and generalization, using a randomized controlled between-subject design. Exercise is well known to benefit mental health (22, 23) and can modulate fear memory processes (15). To date, most human research has focused on acute exercise in the context of fear extinction, generally enhancing consolidation and recall of extinction memories (24), and pointing to reduced threat expectancies following reinstatement (25). To our knowledge, no studies have directly examined whether acute exercise modulates fear generalization, despite its relevance to psychopathology (1, 3, 12). Based on exercise’s general memory enhancing effects, we hypothesized that acute exercise would improve fear learning, with stronger threat-safety discrimination and reduced fear generalization compared to the resting control condition.

## 2 Materials and methods

### 2.1 Participants and study design

Adolescents and young adults aged 16–24 were recruited from the general population and mental health services. After a general screening (exclusion of self-reported major medical and neurological illness, current substance use, current psychiatric medication, autism spectrum disorder, intellectual disability and body mass index > 30), the presence of depressive, anxiety and psychotic symptoms was assessed for group allocation. The following pre-defined cut-offs were selected to capture both subclinical and early clinical symptom levels, consistent with prior studies: ≥ 11 on the Beck Depression Inventory (BDI) (26–28), ≥ 40 on the State Trait Anxiety Inventory (STAI) (29, 30), and ≥ 5 positive answers or ≥ 8 distress score on the Prodromal Questionnaire-16 (PQ-16) (31). Given that mental health symptoms in youth often span multiple symptom dimensions (5), we adopted a transdiagnostic approach to better capture the this early-stage symptom expression. Participants were allocated to the symptom group if they scored above the cut-offs for at least two symptom dimensions. Participants in the healthy control group scored below all cut-offs. The presence of childhood adversity was also assessed using a modified version of the Juvenile Victimization questionnaire (JVQ) (**Supplementary Material**).

The study consisted of two sessions. In session 1, participants performed a maximal cardiopulmonary exercise test (CPET, protocol in **Supplementary Material**) to measure the maximal oxygen consumption (VO_2_max). In addition, participants completed a reassessment of psychiatric symptoms to obtain recent scores. Session 2 took place within 10 days of session 1. In session 2, participants first completed either the acute exercise intervention or rest condition, which was immediately followed by the fear conditioning task. The study was approved by the Local Ethical Committee UZ/KU Leuven (S62702) and performed according to the Declaration of Helsinki. Participants or one of their legal guardians in case of minors provided written informed consent. Participants received monetary compensation for their participation.

### 2.2 Acute exercise intervention

Participants were randomized to either 10 minutes of moderate-intensity cycling (Kettler C8) or rest sitting on the ergometer, using RedCap (32) with permuted block randomization and stratified according to the presence of psychotic symptoms. The individual moderate-intensity exercise level was defined as 50% of their VO_2_max. Participants wore a Polar H10 heart rate monitor and the cycling load was adjusted to maintain heart rates between 45 to 60% of their VO_2_max. The mean intervention-to-task interval was 9min18s (SD = 3min19sec).

### 2.3 Fear conditioning paradigm

**Figure 1:**
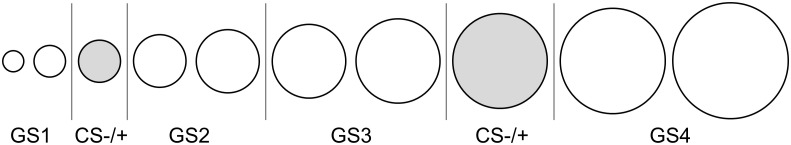
Conditioning and generalization stimuli. The third and eighth circles were counterbalanced to be the CS- or CS+. During acquisition, only the CS- and CS+ were shown. During generalization, all 10 circles were shown.

Fear learning and generalization were measured using a classical fear conditioning task programmed in Python (PsychoPy package) (33, 34). The task uses 10 rings of gradually increasing size (15% difference, diameter from 2 to 4.70 inches) (adapted from Lissek et al., 2008) (**Figure 1**). The third and eighth circles served as the conditioned stimuli, counterbalanced between participants to be the CS- or CS+ (condition 0 or 1), which allows investigation of both danger and safety learning, as well as generalization effects on both sides. A mild electrical shock was used as the unconditioned stimulus (US) and administered to the non-dominant wrist (DS7A electrical stimulator, Digitimer, Welwyn Garden City, UK). Before the experiment, the intensity of the US was individually calibrated. An initial brief 100 ms electrical shock of 2 mA was given and gradually increased with 2 mA. After each shock, participants reported the perceived intensity on a 5-point Likert scale. The final intensity was defined as ‘unpleasant, but not painful’ corresponding to a score of 4 out of 5, or the predetermined maximum current of 24 mA.

The task consisted of three phases (habituation, acquisition and generalization), during which circles were presented on a computer monitor (27-inch). Participants were instructed to learn to predict when they would receive an electrical shock. Fear learning was also assessed physiologically with electromyography (EMG), where a startle probe (40 ms, 95 dB, near-instantaneous rise time) was given, to evoke a fear-potentiated startle response, during half of the trials and a quarter of the intertrial intervals. Unfortunately, due to a technical malfunction in the trigger system, the recorded data could not be reliably analyzed and were therefore excluded. For every trial, participants were shown a fixation cross in the middle of the screen (1 sec), followed by a stimulus presentation (8 sec). Two seconds after the stimulus appeared, a rating scale was shown. Participants indicated their expectancy for receiving the US following that circle on a 10-point Likert scale (1 = definitely no shock, 10 = definitely a shock). The scale disappeared when an answer was given or at stimulus offset in case of a non-response. The intertrial interval was 2.2 sec or 3.2 sec for trials with an intertrial startle probe. During the habituation phase, the CS+ and CS- were shown each four times without the US. Secondly, during acquisition, CS+ and CS- were shown eight times each, with the CS+ paired with a shock at 75% reinforcement rate. In the generalization phase, the CS+ and CS- were shown eight times each, with in addition each of the GSs shown four times. CS+ reinforcement rate was 50%. All trials were semi-randomized with maximally two identical trials shown in a row and clustered within four blocks of 12 trials (2 CS+, 2 CS- and 1 of every GS). Afterwards, participants were asked about their CS-US contingency awareness. Their responses were classified as aware, uncertain or unaware by two researchers. Finally, participants completed post-experimental questions on US expectancy, valence, fear, and arousal level for all the CSs and GSs. A 10-point Likert scale was used to assess US expectancy and fear, while a self-assessment manikin (SAM) scale was used for valence and arousal (35).

### 2.4 Perceptual discrimination paradigm

To control for differences in perceptual discrimination ability (36), participants completed a perceptual discrimination task. This was completed after the fear conditioning task to ensure that the exercise manipulation was always performed directly before the fear conditioning. Participants were presented with two sequential circles and asked to indicate whether these were identical or different. During each trial, a fixation cross was presented in the center of the screen (2 sec), then the first circle (1 sec), followed by a blank screen (2 sec) and the second circle (1 sec). The task consisted of 10 pairs of identical circles, four pairs of CS- and CS+, and 32 pairs comparing the GSs to the CS- and CS+.

### 2.5 Statistical analysis

Statistical analyses were conducted in RStudio (version 4.1.2) (37). Data were analyzed with linear multilevel models with a random intercept (R package lmerTest, version 3.1.3) (38). *Post hoc* tests were performed using the R package emmeans (version 1.8.2) with FDR correction. Three participants (one healthy control, two with symptoms) were excluded from analyses (two due to pressing wrong buttons, one due to technical error). For both the habituation and generalization phase, one extra participant from the symptom group was excluded due to a technical error. The GSs were clustered into four categories (GS1 – GS4) (39). The *a priori* defined covariates age, sex and VO_2_max, and their interactions with stimulus were included in all models. Primary analyses focused on US expectancy ratings as outcomes. Although physiological fear-potentiated startle data were collected, a technical malfunction resulted in substantial data loss, preventing reliable analysis.

Primary analyses focused on US expectancy as an outcome. Separate linear multilevel models were fitted for each phase of the task and included the fixed effects group, intervention, stimulus, trial, condition and contingency, as well as the interactions group x stimulus, intervention x stimulus and contingency awareness x stimulus. Details on the habituation phase can be found in **Supplementary Material.** In the acquisition model, the interaction trial x stimulus was additionally included to account for the learning effect. To investigate the shape of the generalization gradient, we conducted a trend analysis using a linear multilevel model including variables to test for linear, quadratic and cubic trends across stimuli. Besides group differences, we conducted dimensional analyses to examine how continuous variation in symptom severity was associated with threat-safety discrimination and fear generalization. These analyses were restricted to the symptom group, as the healthy control group was selected to have low levels of psychiatric symptoms and childhood adversity, resulting in limited variability. In these models, the group variable was replaced with continuous scores for depressive (BDI), anxiety (STAI), and psychotic (PQ-16) symptoms. Additionally, we accounted for childhood adversity by including the interaction stimulus x childhood adversity severity, which was derived from the Juvenile Victimization Questionnaire.

Perceptual discrimination accuracy was assessed using linear regression models with general perceptual discrimination accuracy and accuracy per stimulus type (CSs and GSs) as outcome variables. Independent variables included group, intervention, contingency, and *a priori* defined covariates (age, sex and VO_2_max). The influence of perceptual discrimination on generalization was assessed by adding mean CS+ discrimination and general accuracy, both interacted with stimulus, to the generalization model.

Participant’s response times and post-experimental ratings were evaluated in separate models (**Supplementary Material**). A sensitivity analysis for US expectancy was conducted excluding 18 participants who did not reach the threshold for the electrical shock before reaching the maximum amount (**Supplementary Material**).

## 3 Results

### 3.1 Sample

A total of 124 adolescents and young adults took part in the study, of whom 121 (60 healthy controls and 61 with symptoms) could be included for analysis (**Table 1**). The symptom group was on average slightly younger (*t*_(118)_ = 3.19, *p* = 0.002). Most participants in the symptom group scored above the cut-offs on all three symptom dimensions or had a combination of anxiety and depressive symptoms (**Supplementary Table 1**). The selected US intensity was on average 13.95 mA (SD = 6.33). The majority of participants were aware of the CS-US contingency (62.81% aware, 22.31% uncertain, 14.88% unaware) and there was no significant group difference (*t*_(2)_ = 4.06, p = 0.13). The sample characteristics of the intervention groups can be found in **Supplementary Table 2**.

**Table 1:** Demographics and measures.

Variable	Scale	Healthy control	Symptoms	*t*/*X*²(DF)	*p*
Sample	-	60	61	-	-
Intervention	exercise/rest	32/28	32/29	-	-
Age	16–24 years	21.22 ± 1.94	20.03 ± 2.14	3.19 (118)	**0.002**
Sex	% females	65.00%	72.13%	0.42 (1)	0.52
Ethnicity	% Caucasian	98.33%	96.72%	0.99 (2)	0.61
Students	% students	93.33%	100%	2.38 (1)	0.12
Body Mass Index	-	21.78 ± 2.51	22.46 ± 3.36	-1.28 (111)	0.20
VO2 max/kg	-	41.17 ± 7.13	38.72 ± 8.96	1.65 (112)	0.10
Depressive symptoms (BDI)	0 – 63	2.53 ± 2.71	19.43 ± 10.27	-12.41 (68)	**< 0.0001**
Anxiety symptoms (STAI)	20 - 80	30.27 ± 5.20	53.38 ± 10.53	-15.34 (88)	**< 0.0001**
Psychotic symptoms (PQ-16)	Symptoms score 0 - 16 Distress score 0 - 48	0.93 ± 1.34 0.47 ± 0.96	4.82 ± 3.29 6.15 ± 6.17	-8.53 (80) -7.10 (63)	**< 0.0001** **< 0.0001**
US intensity	mA	14.57 ± 5.98	13.34 ± 6.65	1.06 (118)	0.29
Contingency	Aware Uncertain Unaware	41 (68.33%) 14 (23.33%) 5 (8.33%)	35 (57.38%) 13 (21.31%) 13 (21.31%)	4.06 (2)	0.13

The scale indicates the theoretical scale of the questionnaires. Groups were compared using unpaired Welch t-tests (mean ± SD) or the chi-squared test for categorical data (%). Cardiorespiratory fitness level is defined as the VO2max divided by their weight (kg). Two participants had no VO2max data, one due to a suboptimal cardiopulmonary test and one due to technical malfunction. BDI, Beck Depression Inventory; STAI, State Trait Anxiety Inventory; PQ-16, Prodromal Questionnaire 16. Bold values indicate statistical significance (p < 0.05).

### 3.2 US expectancy

#### 3.2.1 Acquisition

**Figure 2:**
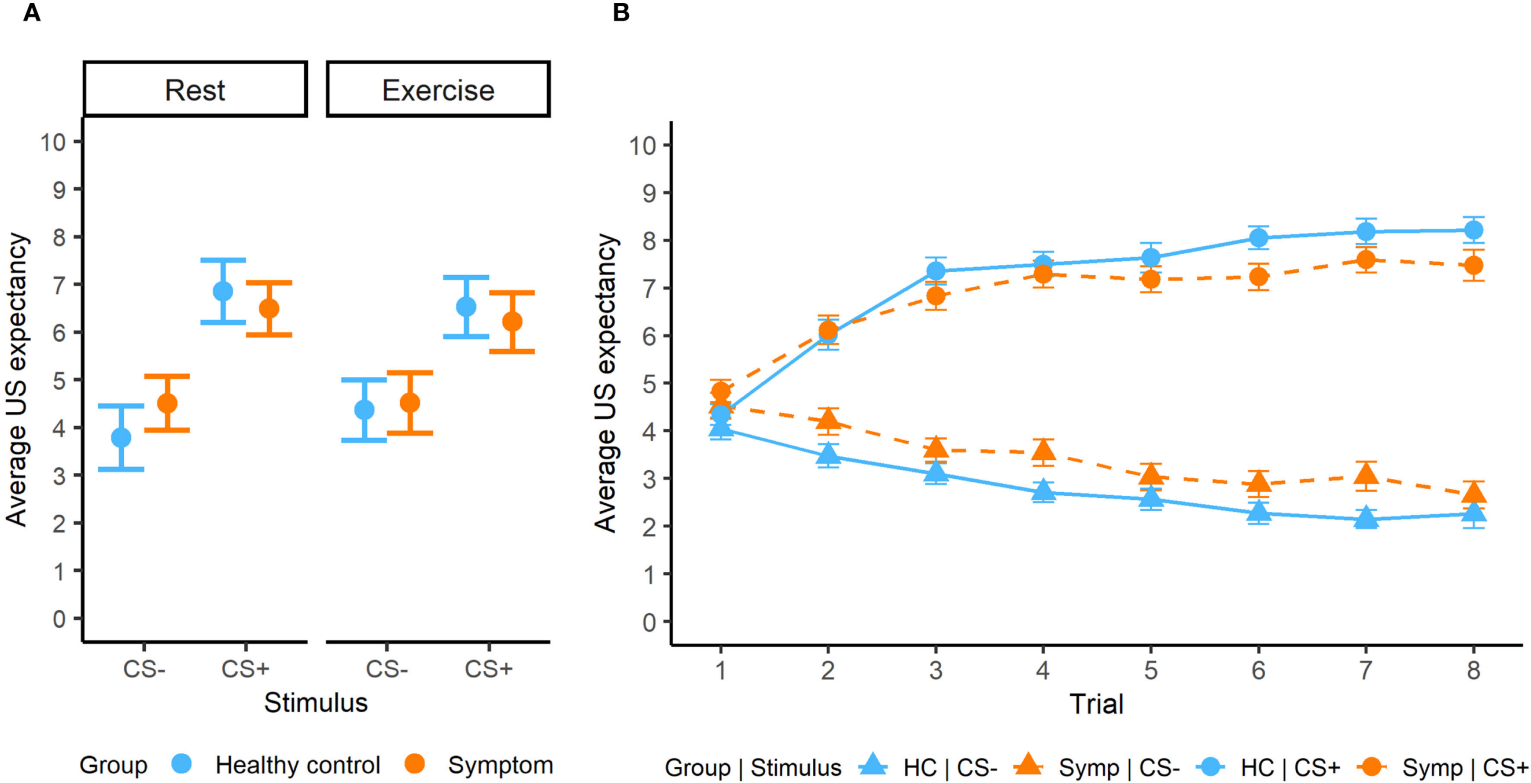
**(A)** Average US expectancy ratings during the acquisition phase for both groups and interventions**. (B)** Learning effect during acquisition phase.

In acquisition, we observed significant interactions between stimulus x group (β = -0.52, SE = 0.19, *p* = 0.007) and stimulus x intervention (β = -0.52, SE = 0.29, *p* = 0.005). We observed adequate threat-safety discrimination with significantly lower US expectancy values for the CS- compared to the CS+ across groups and interventions (all *p* < 0.001), but no significant differences between the groups or interventions (**Figure 2A**). The trial x stimulus interaction was significant (β = 0.33, SE = 0.02, *p* < 0.0001), showing a learning effect (**Figure 2B**). Furthermore, older participants gave significantly lower US expectancy ratings to the CS- (β = 0.27, SE = 0.10, *p* = 0.005, 95% CI [-0.44, -0.08]), but not for the CS+. We observed a significant interaction of stimulus with VO_2_max (β = 0.20, SE = 0.09, *p* = 0.03), but this effect did not survive FDR-corrected *post hoc* tests ([-0.38, 0.03]).

While no significant associations with continuous psychiatric symptom scores could be detected in the dimensional analysis, we observed a significant stimulus-dependent association with childhood adversity (β = 0.13, SE = 0.05, *p* = 0.02), with increased expectancy for the CS+ in association with increased adversity severity ([0.01, 0.20]). The slope was not significant for the CS- ([-0.11, 0.07]).

#### 3.2.2 Generalization

**Figure 3:**
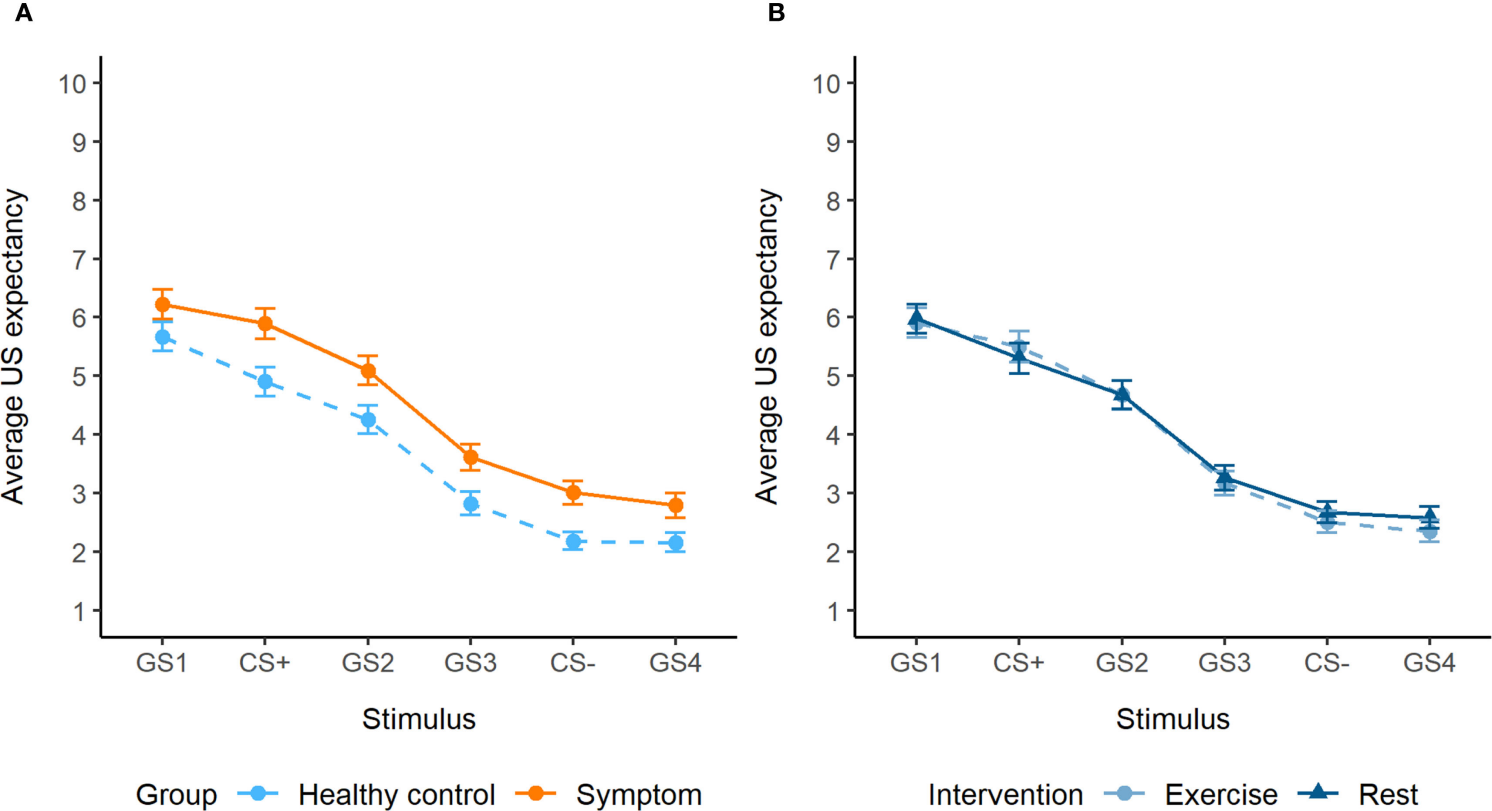
**(A)** Generalization gradients of the symptom group and healthy control group, with overall increased expectancy ratings in the symptom group (significant for the GS1, CS+, GS2 and CS-). **(B)** Generalization gradients of the exercise and rest intervention, showing no significant differences.

During generalization, a significant interaction between group and stimulus was observed (F_5,5454_ = 3.55, *p* = 0.003). *Post hoc* tests showed significantly higher US expectancy ratings in the symptom group compared to the healthy control group for the GS1 (β = -0.64, SE = 0.26, *p* = 0.01), CS+ (β = -1.06, SE = 0.26, *p* = 0.0001), GS2 (β = -0.68, SE = 0.26, *p* = 0.01) and CS- (β = -0.52, SE = 0.26, *p* = 0.048) (**Figure 3**). While there was an interaction between intervention and stimulus (p = 0.02), *post hoc* tests were not significant. Generalization results were not influenced by differences in perceptual discrimination ability (**Supplementary Material**). Trend analysis showed a cubic trend for stimulus (β = -3.35, SE = 0.46, *p* < 0.0001), which did not differ between the groups, indicating a similar generalization gradient.

Furthermore, older participants displayed lower US expectancy values for GS3 (95% CI [-0.99, -0.19]) and GS4 ([-0.84, -0.04]). There was a stimulus-dependent effect of sex (F_5,5454_ = 5.15, *p* < 0.0001), but this did not remain significant in *post hoc* tests. Overall, US expectancy decreased across trials (β = -0.033, SE = 0.002, *p* < 0.0001).

**Figure 4:**
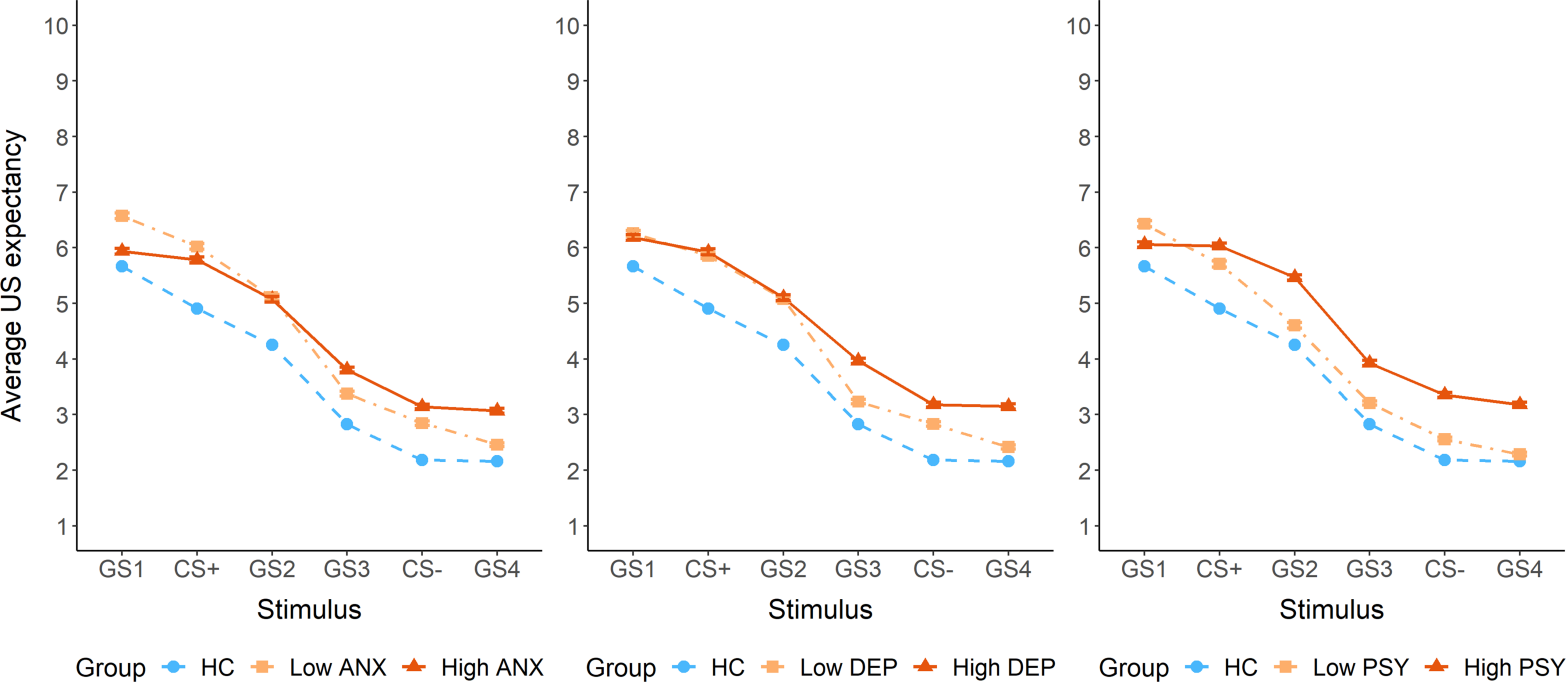
US expectancy ratings during the generalization phase, plotted separately for high and low symptom groups based on a median split for anxiety symptoms (State Trait Anxiety Inventory – STAI), depressive symptoms (Beck Depression Inventory – BDI), and psychotic symptoms (Prodromal Questionnaire – PQ-16). ANX = anxiety symptoms, DEP = depressive symptoms, PSY = psychotic symptoms.

The dimensional analysis showed significant positive, stimulus-dependent associations of US expectancy with depressive (F_5,2697_ = 3.64, *p* = 0.003), anxiety (F_5,2697_ = 8.12, *p* < 0.0001) and psychotic symptoms (F_5,2697_ = 3.06, *p* = 0.009), but not with childhood adversity (*p* = 0.2). *Post hoc* tests detected significant positive slopes for the GS1 ([0.006, 0.13]) and GS3 ([0.003, 0.07]) in association with depressive symptoms, and for the GS2 ([0.001, 0.28]) in association with psychotic symptoms. The slopes in association with anxiety symptoms were not significantly different from zero. For visualization purposes, we plotted US expectancy as a function of high and low symptom scores based on a median split (**Figure 4**). This categorical grouping was not part of the statistical analysis plan and should be interpreted as exploratory, serving only to aid visual interpretation of the generalization patterns. The plots suggest that higher anxiety and psychotic symptoms are particularly associated with a reduced peak at GS1 compared to lower symptom levels. In contrast, higher depressive symptoms were primarily associated with elevated US expectancy on the safety side of the gradient.

#### 3.2.3 Contingency awareness

**Figure 5:**
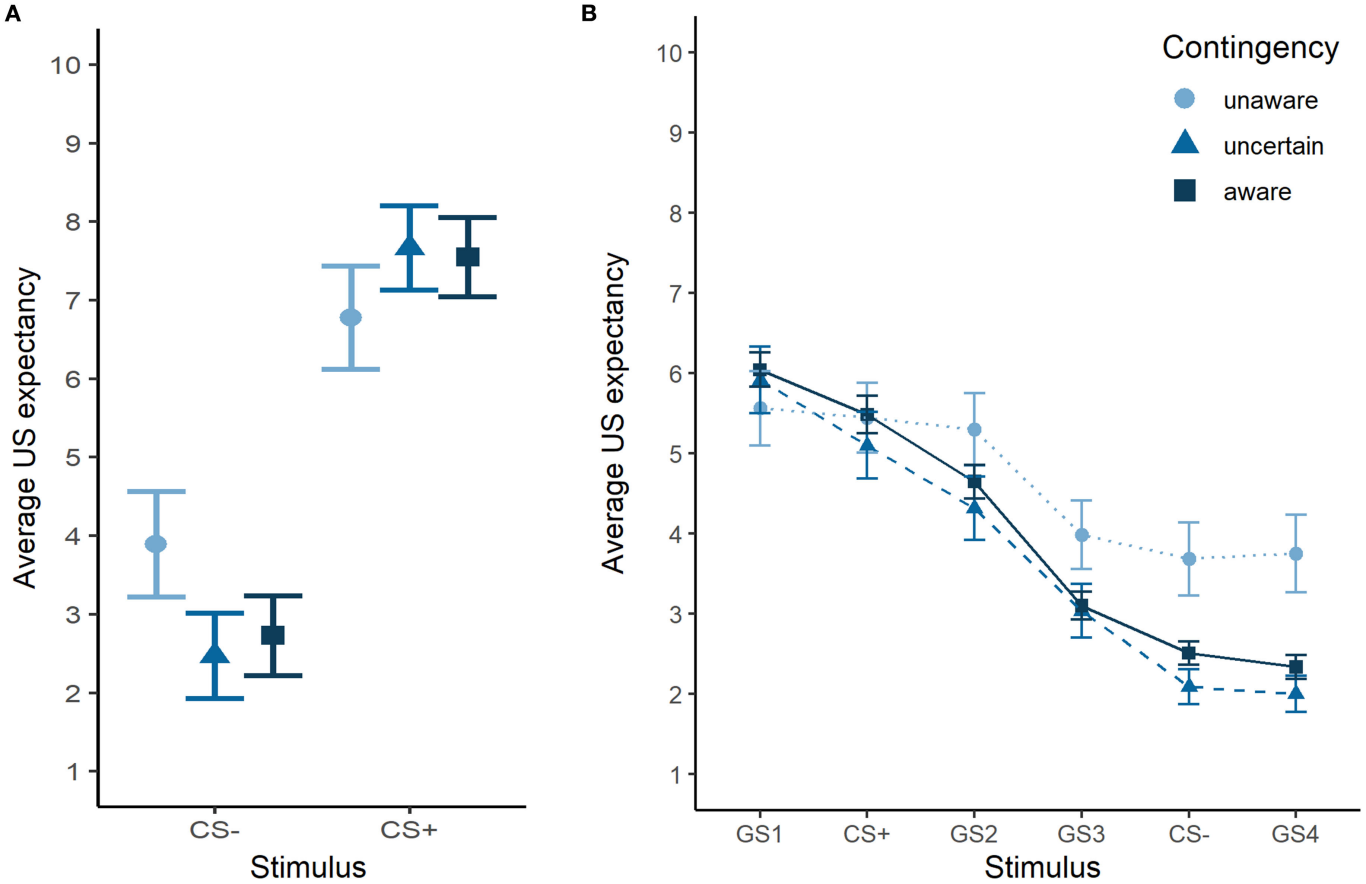
**(A)** Average US expectancy ratings during the acquisition phase for each contingency awareness group **(B)** Generalization gradient for each contingency awareness group.

During acquisition, a significant interaction between contingency awareness and stimulus was found (F_2,1745_ = 32.78, *p* < 0.0001), with less extreme values for CS- and CS+ in the unaware participants compared to the uncertain and aware participants (all *p* < 0.01), but no significant difference between uncertain and aware participants (**Figure 5A**). Nevertheless, the unaware participants acquired threat-safety learning with significantly different US expectancy ratings for the CS+ and CS- (β = -2.09, SE = 0.25, *p* < 0.0001). Also in the generalization phase there was a significant stimulus-dependent effect of contingency awareness (F_10,5454_ = 8.72, *p* < 0.0001), with significant differences for the GS3 (unaware vs aware β = 0.89, SE = 0.36, *p* = 0.047), the CS- (unaware vs uncertain β = 1.38, SE = 0.42, *p* = 0.003; unaware vs aware β = 1.12, SE = 0.36, *p* = 0.003) and the GS4 (unaware vs uncertain β = 1.68, SE = 0.42, *p* = 0.0001; unaware vs aware β = 1.48, SE = 0.36, *p* = 0.0001) (**Figure 5B**). A sensitivity analysis was conducted excluding the unaware participants (*n* = 18), but this did not affect the results (**Supplementary Material**).

## 4 Discussion

### 4.1 Increased overall fear instead of overgeneralization in the symptom group

Adequate threat-safety discrimination was observed during acquisition across groups, based on subjective US expectancy ratings. Although the symptom group showed slightly less distinct US expectancy values (e.g., higher for CS−, lower for CS+), these differences were not statistically significant, suggesting intact threat-safety learning. Both groups exhibited similar generalization gradients, and we found no evidence of fear overgeneralization. Controlling for perceptual discrimination ability did not change these results.

These findings contrast with our hypotheses and prior literature linking anxiety and stress-related disorders to increased fear to safety cues and overgeneralization of fear (3, 6, 8). Several factors may explain why we did not observe the expected group differences. First, our sample comprised youth with subclinical or early-stage psychiatric symptoms rather than a clinical population; stronger effects may emerge in individuals with more severe symptomatology. Nevertheless, fear generalization has been proposed as a dimensional phenotype (3), and a meta-analysis showed increased generalization even in healthy individuals high in anxious traits (10). Reduced threat-safety discrimination has similarly been linked to elevated trait anxiety (40), and longitudinal work suggests that impaired CS-/CS+ discrimination can predict future anxiety (9). However, findings are mixed; other studies have failed to observe associations between trait anxiety and either acquisition or generalization processes (41, 42), underscoring the complexity of these processes. Notably, anxiety-related increases in threat responding may reflect differences in intolerance of uncertainty, which may shape threat-safety discrimination as shown in prior work using skin conductance (43).

Another consideration is the heterogeneity of our transdiagnostic sample, which included young people with anxiety, depressive and psychotic symptoms. While reduced discrimination and fear overgeneralization have been most consistently linked to anxiety, aberrant threat processing may not be exclusive to anxiety symptomatology. For instance, enhanced generalization was observed in youth with subclinical depressive and psychotic symptoms, but only in those with high levels of childhood maltreatment (1). It is therefore possible that the symptom overlap, although typical at this developmental stage, may have obscured more specific alterations in threat processing at the group level.

Importantly, the symptom group displayed overall elevated US expectancy ratings (significant for GS1, CS+, GS2, and CS-) compared to the healthy controls during generalization. This occurred despite intact discrimination abilities during acquisition, suggesting a cognitive-affective bias in interpreting ambiguous stimuli as threatening rather than a learning impairment. This points to a general threat expectancy bias in the symptom group, aligning with prior work linking a cognitive threat bias to not only anxiety, but depression and psychosis as well (1). Notably, this increased US expectancy emerged only during generalization and not during acquisition. The generalization phase introduces greater ambiguity by means of a new context, novel GSs and a lower reinforcement rate, reducing predictability and increasing uncertainty. This may be particularly distressing for youth with mental health symptoms due to heightened intolerance of uncertainty, leading to amplified fear. This pattern underscores the importance of also considering overall levels of fear responding, as this may reveal early threat-related biases not captured by gradient shape alone. Indeed, Stegmann et al. emphasized the importance of examining mean fear responses alongside generalization gradients (44), and a recent study observed similar overall increased US expectancy ratings in adolescents with anxiety disorders (45).

Although more research is needed, elevated US expectancy may represent an early-stage vulnerability marker in threat processing during the transitional period between late adolescence and young adulthood, possibly preceding more specific alterations in discrimination or generalization. While our transdiagnostic approach fits with the characteristics of our population, its heterogeneity may mask similarities or distinctions in threat processing patterns tied to individual symptom dimensions – a question we explore further in our dimensional analysis.

### 4.2 Symptom-specific alterations in fear generalization patterns

The dimensional analysis examined whether specific psychiatric symptoms were differentially associated with alterations in fear learning and generalization patterns. Although no significant associations emerged during acquisition, anxiety, depressive and psychotic symptoms significantly influenced US expectancy during generalization. *Post hoc* analyses showed increased US expectancy only for GS1 and GS3 in association with depressive symptoms, and for GS2 in association with psychotic symptoms. While statistical effects were modest, likely reflecting the smaller sample size of the symptom group and applied FDR correction, these analyses suggest subtle symptom-specific variations in US expectancy during generalization.

Notably, childhood adversity was unrelated to US expectancy during generalization but was associated with heightened CS+ responding during acquisition. This finding contrasts with the majority of previous literature finding reduced threat-safety discrimination in individuals with childhood adversity (46). Although not the focus of this study, this distinction does suggest that adversity may primarily impact initial fear learning, whereas psychiatric symptoms may affect how threat generalizes.

To explore symptom-specific generalization patterns, we visually examined median-split plots. Importantly, these exploratory visualizations were not part of the main statistical analysis and should be interpreted cautiously. For anxiety and psychotic symptoms, we observed increased US expectancy on the safety side, alongside decreased discrimination between the CS+ and GS1 (i.e. perceptually most extreme but unreinforced stimulus). While healthy controls displayed an extremity bias or peak shift, with highest expectancy at GS1, this pattern was lost in those with high anxiety and psychotic symptoms. This loss of differentiation may reflect a diminished capacity to discriminate subtle ambiguities. Alternatively, intolerance of uncertainty may prompt avoidance of nuanced threat evaluation, resulting in a more uniform response across stimuli (47, 48). Depressive symptoms appeared to affect only the safety side of the gradient, with higher symptom severity linked to increased US expectancy for safety cues. This pattern may reflect impairments in reward processing, leading to a pessimistic bias or deficits in fear inhibition. This aligns with prior work showing increased US expectancy of safe stimuli in young people with greater anhedonia-apprehension (12).

Together, these findings suggest that elevated US expectancy and altered generalization gradients may reflect distinct processes. While group comparisons capture broad vulnerability, dimensional analyses point to symptom-specific variations that may signal early differentiation within a shared risk profile. However, statistical effects were limited, and interpretation should be cautious due to the reduced power from sample size and stratification. Exploratory visual trends nonetheless suggest symptom-related differences that merit further investigation. Future studies with larger samples could apply more sensitive analytical techniques (e.g., multivariate pattern analysis, latent profile modelling) to better characterize these patterns, which could possibly inform early detection and targeted interventions.

### 4.3 No acute exercise effects on fear acquisition or generalization

While prior studies have primarily examined exercise effects on fear conditioning and extinction, this study is, to our knowledge, the first to investigate whether acute exercise influences fear generalization. Contrary to our hypotheses, the intervention did not affect threat–safety learning or fear generalization, based on subjective US expectancy ratings, in either healthy individuals or those with elevated psychiatric symptoms. Although animal studies link acute exercise to memory enhancement (15, 49, 50), humans studies have mostly focused on extinction paradigms, where exercise post-extinction reduced fear (25, 51, 52). Fear generalization, however, depends not only on memory strength but also on the specificity of learned threat associations. Interestingly, a recent study found that 20 minutes of vigorous exercise after extinction enhanced generalization to perceptually similar stimuli (53). Exercise might enhance the salience or consolidation of the original threat memory, resulting in more generalization. Our null findings may reflect that the 10-minute moderate-intensity intervention was insufficient in duration or intensity (54). Future research should investigate how exercise duration, intensity and timing affect fear generalization.

### 4.4 Decreased threat-safety discrimination and overgeneralization of fear in contingency unaware participants

Participants unaware of the CS–US contingency showed significantly reduced threat–safety discrimination compared to uncertain and aware participants. This likely contributed to their flatter generalization gradient, characterized by significantly higher US expectancy ratings for the safety cue and surrounding stimuli during generalization. The existence of contingency-unaware fear conditioning remains debated, with limited supporting evidence (55). Nonetheless, unaware participants in our study still exhibited a significant CS+/CS– differentiation, albeit reduced, suggesting either implicit learning or difficulty articulating the association. Of note, in our sample, 62.81% of participants were classified as aware, which aligns with the complexity of the task, including the number of stimuli and the reduced reinforcement rate during generalization. Variability in how contingency awareness is assessed hinders strong conclusions and highlights the need for standardized procedures in future work.

### 4.5 Fear learning in adolescents and young adults

Prior research on age-related differences in fear learning is mixed, but often shows decreased threat-safety discrimination and increased fear generalization in younger children (56, 57). However, existing studies predominantly focus on either adults or young children, leaving a gap in adolescent to young adult populations. We selected a 16-24-year-old sample to capture a developmental period of heightened vulnerability for the emergence of mental health problems. Within this range, older participants showed decreased US expectancy ratings for the CS- during acquisition and for the GS3 and GS4 during generalization, suggesting enhanced safety learning. Post-experimental ratings supported this, indicating increased valence but decreased fear, arousal and US expectancy, as age increased. This pattern of results aligns with the idea that older individuals in this age range may have developed more effective safety learning or a better ability to differentiate between threat and safety. While this age span enables the examination of threat processing across a clinically relevant transition period, ongoing brain maturation beyond 18 years warrants caution when comparing these findings to studies restricted to adolescents or adults. These findings contribute to the growing literature on age-related differences, emphasizing the importance of examining fear conditioning across developmental stages.

### 4.6 Limitations

This study has several limitations. First, the heterogeneity of the sample with mixed psychiatric symptoms poses challenges when comparing with other clinical samples. Additionally, participants were predominantly highly educated and Caucasian, limiting generalizability. Second, using individually selected shock intensities for the US led to 18 participants not reaching the predefined threshold, resulting in a milder subjective experience of the shock. Although a sensitivity analysis was conducted, this may still have influenced results. While sex was included as a covariate, further exploration of sex differences and hormonal influences is necessary considering their relevance in affective disorders. Third, due to design constraints because of the intervention, the fear conditioning and perceptual discrimination tasks were not counterbalanced, which may have increased fatigue or reduced engagement during the latter task. This could have limited variability and contributed to the null association between perceptual discrimination and fear conditioning performance. An additional limitation is that, although US-expectancy ratings provide a validated measure of subjective fear learning (58), we were unable to analyze physiological indices of fear learning (fear-potentiated startle) due to a technical malfunction in the trigger system. While we prioritized the accuracy and reliability of the data focusing on US expectancy outcomes, which captures conscious threat appraisal important in translational and clinical contexts, objective measures would have complemented these findings and strengthened conclusions regarding implicit fear responses. Furthermore, while dimensional analyses were pre-specified and informative, the smaller sample size limited statistical power to detect stimulus-specific effects. Despite trends suggesting symptom-specific variations in generalization patterns, these results should therefore be interpreted with caution and further research is necessary. Lastly, longitudinal studies, using larger sample sizes and incorporating psychophysiological measures, are needed to better understand causality and directionality of the association between fear generalization and psychiatric symptoms.

## 5 Conclusion and future perspectives

In conclusion, youth with early-stage psychiatric symptoms did not show expected deficits in discrimination or overgeneralization but exhibited overall elevated threat expectancy during generalization, as indexed by US expectancy ratings. This may reflect an early-stage vulnerability in threat processing. Dimensional analysis point to modest symptom-specific differences in generalization patterns, underscoring the importance of examining continuous symptom severity, alongside group-based comparisons in understanding fear learning during the critical transition from late adolescence to early adulthood. Future research should use larger samples to validate these effects and explore longitudinal trajectories on how these patterns evolve with symptom progression. Additionally, further work is needed to identify exercise characteristics that may influence fear learning, to better assess its utility as an intervention.

## Data Availability

The datasets presented in this article are not readily available because the data that support the findings of this study are available on request from the corresponding author (LJ). The pseudo-anonymized data are not publicly available due to the sensitive nature of the clinical data and privacy of research participants. Data are located in controlled access data storage at KU Leuven. Data sharing is possible after submitting a formal project outline, having a formal data sharing agreement and when the request does not interfere with in-house research plans. Co-authorship for data collection and processing is required. Requests to access the datasets should be directed to Lise Jennen, lise.jennen@kuleuven.be.
