# Supplementary material for: Fear learning and generalization in youth with early-stage transdiagnostic psychiatric symptoms and the impact of acute exercise
Source: Front Psychiatry. 2025 Sep 19;16:1657470. doi: 10.3389/fpsyt.2025.1657470 (PMC12491019; doi:10.3389/fpsyt.2025.1657470)
Supplement: Supplementary file 1 [file SupplementaryFile1.docx]

Supplementary Material

# Childhood adversity severity score

Exposure to adverse events was assessed with a modified version of the Juvenile Victimization Questionnaire 2nd revision (Adult Retrospective Form) (JVQ-R2), supplemented with five items of the Emotional Neglect subscale of the Childhood Trauma Questionnaire (CTQ) (Bernstein & Fink, 1998). The assessment covered 33 dichotomous (yes/no) questions across seven categories: bullying/peer victimization, physical abuse, emotional abuse, sexual abuse, physical neglect, emotional neglect, and domestic violence. For each endorsed experience, participants reported its frequency (1 = once, 2 = seldom, 3 = sometimes, 4 = often, 5 = very often). A continuous measure of childhood adversity was calculated by summing the mean frequency scores for each adversity category.

# Incremental cardiopulmonary test protocol

The maximal oxygen uptake or VO_2_max was measured with an incremental cardiopulmonary exercise test on an ergometer (Vyntus CPX, Vyair medical, USA). A step-wise protocol was used with 20+20 or 50+25 W/min according to the participant’s estimated cardiorespiratory fitness level (Balady et al., 2010). Maximal effort was defined as when the participant terminated due to exhaustion, dyspnea, pain or tiredness in the legs, and a peak respiratory exchange rate >1.10. Afterwards, participants cycled for 3 min at 25 W. VO_2_max, was defined as the highest obtained average oxygen consumption over 10 seconds during the test (Guazzi et al., 2012; Mezzani, 2017). The individual moderate-intensity exercise level was defined as 50% of their VO_2_max.

# Frequency of symptoms

Based on screening, 61 participants were allocated to the symptom group, scoring above the cut-offs for at least two of the three symptom dimensions. The symptom frequencies and combinations can be found in **Supplementary Table 1**. During the first session, the symptom questionnaires were repeated to obtain recent levels. Differences in frequency between the screening and session may be due to the additional time between screening and session due to practical constraints, as well as the still unstable nature of symptoms in this stage and population. The distribution of the symptom scores, taking into account the theoretical scales of the questionnaires, can be found in **Supplementary Figure 1**.

**Supplementary Table 1.** Frequencies of symptom dimensions in the symptom group

| # Dimensions | Symptom dimensions | Screening | Session |
| --- | --- | --- | --- |
| 0 | - | 0 | 2 |
| 1 | Anxiety | 0 | 5 |
|  | Depression | 0 | 1 |
|  | Psychosis | 0 | 1 |
| 2 | Anxiety + depression | 35 | 19 |
|  | Anxiety + psychosis | 2 | 4 |
|  | Depression + psychosis | 0 | 1 |
| 3 | Anxiety + depression + psychosis | 24 | 28 |

Legend: Number of participants scoring on the symptom dimensions above the predefined cut-offs for each individual symptom dimension and the combinations, reported separately for the screening and the first experimental session. Anxiety symptoms measured with the State Trait Anxiety Inventory, Depressive symptoms with the Beck Depression Inventory, and psychotic symptoms with the Prodromal Questionnaire 16.


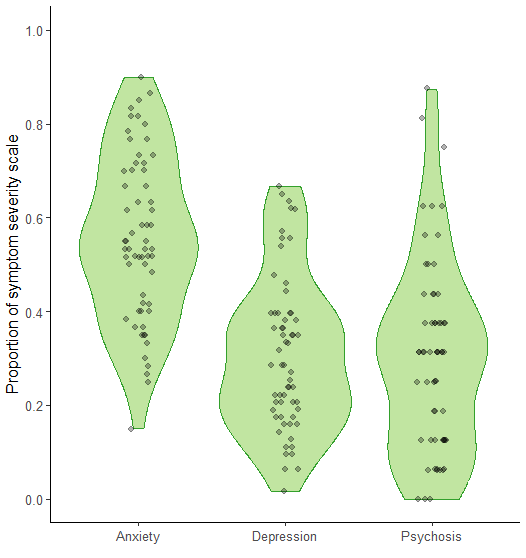

**Supplementary Figure 1.** Distribution of symptom severity scores across participants, expressed as proportions of the respective scale ranges. Depression scores (BDI-II) were divided by 63, anxiety scores (STAI-T) by adjusting for the 20–80 range, and psychotic symptoms (PQ-16) by 16. Each violin plot shows the density of responses, with individual data points overlaid.

# Sample characteristics for the intervention groups

**Supplementary Table 2.** Demographical data for the intervention groups

|  | Scale | Rest | Exercise |
| --- | --- | --- | --- |
| Sample | - | 57 | 64 |
| Group (control/symptoms) | - | 28 / 29 | 32 / 32 |
| Age (years) | 16 - 24 | 20.58 ± 2.05 | 20.56 ± 2.20 |
| Sex (% females) | - | 68.42 | 68.75 |
| Ethnicity (% Caucasian) | - | 98.25 | 96.88 |
| Students (%) | - | 94.74 | 98.44 |
| Body Mass Index | - | 21.64 ± 2.77 | 22.55 ± 3.11 |
| VO2 max/kg | - | 41.17 ± 8.48 | 38.81 ± 7.77 |
| Depression (BDI) | 0 - 63 | 11.84 ± 10.92 | 10.34 ± 11.72 |
| Anxiety (STAI) | 20 - 80 | 43.40 ± 13.57 | 40.59 ± 14.83 |
| Psychosis (PQ-16) | Symptoms score 0 – 16 Distress score 0 - 48 | 3.11 ± 3.35 3.72 ± 6.05 | 2.70 ± 3.03 2.98 ± 4.46 |
| US intensity (mA) | - | 14.04 ± 6.38 | 13.88 ± 6.33 |
| Contingency (%) | Aware Uncertain Unaware | 35 (61.40%) 11 (19.30%) 11 (19.30%) | 41 (64.06%) 16 (25.00%) 7 (10.94%) |

Legend: Cardiorespiratory fitness level is defined as the VO2max divided by their weight (kg). Two participants had no VO2max data, one due to a suboptimal cardiopulmonary test and one due to technical malfunction. BDI = Beck Depression Inventory, STAI = State Trait Anxiety Inventory, PQ-16 = Prodromal Questionnaire 16.

# Habituation phase

The fear conditioning paradigm was started with a habituation phase, where the CS+ and CS- were shown four times each without the US. With US expectancy as an outcome, a separate linear multilevel model was fitted with two-way interactions of group*stimulus and intervention*stimulus, the variables trial and condition (0 or 1) and *a priori* defined covariates age, sex and VO_2_max.

Significantly higher US expectancy ratings were observed further in the task (β = 0.06, SE = 0.02, *p* = 0.002), which might be due to increased uncertainty during this phase that an electrical stimulus might arise after all. Additionally, older participants had significantly lower scores (β = -0.53, SE = 0.18, *p* = 0.004).

# Perceptual discrimination results

No significant associations were found between group, intervention or continuous psychiatric symptom scores with mean perceptual discrimination accuracy. Looking at specific CS or GS perceptual discrimination, we observed borderline significant higher CS+ perceptual discrimination accuracy associated with a higher VO_2_max level (β = 0.03, SE = 0.01, *p* = 0.045). There were no significant associations with symptom scores.

When controlling for CS+ perceptual discrimination and general accuracy in the generalization analysis, the results remained similar. We observed a significant stimulus-dependent effect of general accuracy on US expectancy during the generalization phase (*p* = 0.02), but post hoc tests were not significant.

# Sensitivity analysis: Contingency awareness

18 participants were classified as unaware regarding their contingency awareness. As there were significant differences for the unaware compared to the uncertain and aware participants, during both acquisition and generalization phase, we conducted a sensitivity analysis on a subset excluding the unaware participants. For both the acquisition and generalization phase, the same conclusions can be drawn as in the main analysis.

# Sensitivity analysis: Calibration

A sensitivity analysis excluding the participants (n = 18) that did not reach the predetermined electrical shock intensity before reaching the ethical maximum revealed similar results as the main analysis. Only in the generalization phase, there was no longer a significant difference for the GS2 (*p* = 0.15) and CS- (*p* = 0.18) between the groups.

# Response times

For response times as an outcome, all models included the independent variables group, intervention, stimulus, trial and condition. In all phases of the task, there was a main effect of trial (habituation: β = -0.16, SE = 0.01, p < 0.0001; acquisition: β = -0.02, SE = 0.004, p < 0.0001; generalization: β = -0.005, SE = 0.08, p < 0.0001). There were no significant differences in response times, except for an increased response time for the GS2 in the healthy control group compared to the symptom group during generalization (F_5,5474_ = 2.99, *p* = 0.01; β = 0.22, SE = 0.10, *p* = 0.02). A significant interaction between stimulus and intervention (F_5,5474_ = 2.61, *p* = 0.02) was observed, however, post hoc contrasts were no longer significant. There were no significant associations with dimensional symptom scores.

# Post-experimental ratings

## Main group models

For the post-experimental ratings of US expectancy, valence, arousal and fear, separate linear mixed models were fitted with two-way interactions between group*stimulus and intervention*stimulus, the variable condition, and the a priori defined covariates age, sex and VO_2_max.

Overall, we did not observe any main or interaction effects of group or intervention across the post-experimental ratings. There was a main effect of condition for valence (b = -0.42, SE = 0.17, *p* = 0.02) and arousal (b = 0.44, SE = 0.20, *p* = 0.03). Additionally, we did observe age and sex associations. There was a main effect of age on fear ratings, with significantly overall decreased fear in older participants (b = -0.32, SE = 0.16, *p* = 0.04). For the arousal, fear and US expectancy post-experimental ratings, there was a stimulus-dependent age effect. Older participants displayed decreased arousal (F_5,1034_ = 3.05, *p* = 0.01), which was only significant for the GS2 (95% CI [-0.62, -0.07]), GS3 ([-0.76, -0.21]), CS- ([-0.80, -0.11]) and GS4 ([-0.81, -0.26]) stimuli in post hoc tests. Similarly, older participants displayed decreased US expectancy risk ratings (F_5,1037_ = 3.48, *p* = 0.004), significant for the GS2 ([-0.61, -0.007]), GS3 ([-0.84, -0.24]) and CS- ([-0.85, -0.06]). In contrast, older participants had higher valence (F_5,1025_ = 2.79, *p* = 0.02), significant for the GS3 ([0.12, 0.56]) and GS4 ([0.13, 0.69]). For the sex differences, valence ratings were dependent on sex (F5,1025 = 3.95, *p* = 0.001), with females giving significantly less extreme values to the GS1 (b = -0.86, SE = 0.37, *p* = 0.02) and GS4 (b = 0.86, SE = 0.37, *p* = 0.02), indicating a flatter gradient. A similar trend could be observed with a significant stimulus-dependent effect of sex on arousal (F5,1034 = 2.62, *p* = 0.02), but individual stimulus contrasts in post hoc tests were not significant.

## Dimensional analysis

Associations with individual symptom dimensions were also explored in separate models within the symptom group subset, replacing the group variable with continuous symptom scores (depressive (BDI), anxiety (STAI) and psychotic (PQ-16)).

For valence, we observed a borderline significant interaction of stimulus with psychosis symptoms (*p* = 0.046), with significantly higher valence for the GS1 (95% CI [0.05, 0.28]) and GS2 ([0.04, 0.28]). In addition, there were significant stimulus-dependent interactions of depressive symptoms on US expectancy (*p* = 0.007), arousal (*p* = 0.001) and fear (*p* = 0.01). However, post hoc tests only showed significant positive for the GS2 ([0.002, 0.10]) and GS3 ([0.006, 0.11)] on arousal, but no significant slopes on US expectancy or fear ratings.
